# Supplementary figures and images for: Activation of the aryl hydrocarbon receptor inhibits neuropilin-1 upregulation on IL-2-responding CD4+ T cells
Source: Front Immunol. 2023 Nov 14;14:1193535. doi: 10.3389/fimmu.2023.1193535 (PMC10682649; doi:10.3389/fimmu.2023.1193535)

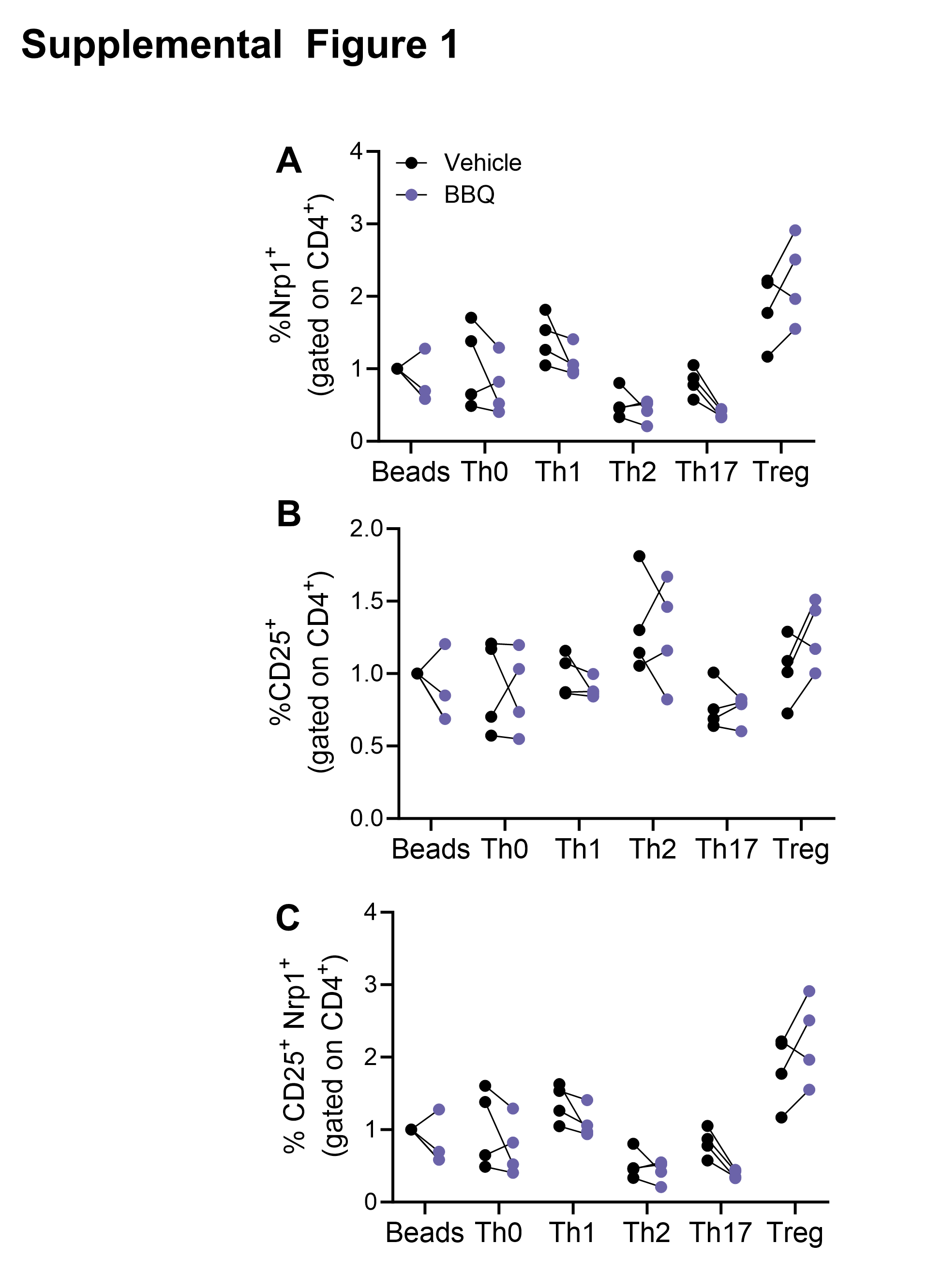

Supplement: Supplementary Figure 1 — CD4+CD25- T cells were isolated using EasySep™ Mouse CD4+ T Cell Isolation Kit (StemCell Technologies; Cat # 19852A) supplemented with biotinolyated anti-CD25 (clone PC61.5; StemCell Technologies; Cat #60009BT.2) from the spleen of NOD mice. Foxp3 depletion was confirmed; 75-80% Foxp3+ CD4+ T cells were removed. Cells were polarized toward Th0, Th1, Th2, Th17, or Treg. All cells were also co-cultured with CD3/CD28 Dynabeads. AhR was activated with 100 nM Cl-BBQ. Cells were incubated at 37°C for 4 days. Cells from WT mice were stained for Nrp1, CD25, and CD4. The percentage of Nrp1+ (A), CD25+ (B), and Nrp1+CD25+ (C) were examined. n=4 mice/cell culture condition. Each data point represents cells from an individual mouse. [file Image_1.tif]
